# Bridging the gap in neonatal resuscitation in Zambia

**DOI:** 10.3389/fped.2022.1038231

**Published:** 2022-12-05

**Authors:** Kunda Mutesu-Kapembwa, Jyoti Lakhwani, Rodgers Gift Benkele, Sylvia Machona, Mwila Sekeseke Shamalavu, Jean Musonda Chintende, Susan Mwila Chisela, Sharon Kapoma, Jackson Mwanza, Wisdom Chelu, Martha Mwendafilumba, Kenneth Kapembwa, Vincent D. Gaertner

**Affiliations:** ^1^ Department of Neonatology, Women and Newborn Hospital, University Teaching Hospitals, Lusaka, Zambia; ^2^ Newborn Support Zambia, Lusaka, Zambia; ^3^ Paediatric Nurses Association of Zambia, Lusaka, Zambia; ^4^ Midwives Association Zambia, Lusaka, Zambia; ^5^ Council of International Neonatal Nurses, Lusaka, Zambia; ^6^ Clinical Anaesthetist Association of Zambia (CAAZ), California, CA, United States; ^7^ Newborn Research, Department of Neonatology, University Hospital and University of Zurich, Zurich, Switzerland; ^8^ Dr von Hauner University Children's Hospital, Ludwig-Maximilians-University, Munich, Germany

**Keywords:** neonatal resuscitation, resource limited, helping babies breathe, algorithm, bag and mask

## Abstract

Neonatal resuscitation has been poorly instituted in many parts of Africa and most neonatal resuscitation algorithms are adapted from environments with abundant resources. Helping Babies Breathe (HBB) is an algorithm designed for resource-limited situations and most other algorithms are designed for resource-rich countries. However, there are neonatal referral centers in resource-limited countries who may provide more advanced resuscitation. Thus, we developed a neonatal resuscitation algorithm for a resource-limited country (Zambia) which considers more advanced interventions in situations where they can be provided. The algorithm described in this paper is based on the Newborn Life Support algorithm from the UK as well as the HBB algorithm and accounts for all situations in a resource-limited country. Most importantly, it focuses on non-invasive ventilation but includes advice on more advanced resuscitation including intravenous access, fluid management, chest compressions and adrenaline for resuscitation. Although intubation skills are included in neonatal training workshops, it is not the main focus of the algorithm as respiratory support equipment is scarce or lacking in most health facilities in Zambia. A home-grown neonatal resuscitation algorithm for a resource-limited country such as Zambia is likely to bridge the gap between limited situations requiring only bag and mask ventilation and better equipped institutions where more advanced resuscitation is possible. This algorithm will be rolled out in all training institutions and delivery facilities across Zambia over the next months.

## Introduction

Neonatal resuscitation has been poorly instituted in many parts of Africa and other limited-resource settings (1). This is mainly due to insufficient instruction in neonatal resuscitation techniques during under- and postgraduate training and lack of tools to carry out neonatal resuscitation.

Most neonatal resuscitation algorithms used in African countries are adapted from environments with abundant resources (2). In most countries, neonatal resuscitation is not standardized, and the algorithms used depend on the training of the individual resuscitator. The majority of the qualified staff have received training in the helping babies breathe (HBB) algorithm (1, 3), while others have been trained using the Resuscitation Council (UK) newborn resuscitation algorithm, frequently employed within the National Health Service (NHS) (4, 5). However, as medical care in Zambia and across many other resource-limited countries largely depends on the individual situation (e.g., rural vs. metropolitan, university hospital vs. small outpost clinic), neither algorithm is truly designed for all situations.

Neonatal mortality is the highest contributor to deaths of under five-year-old children and (6) Zambia continues to record high neonatal deaths due to birth asphyxia, making it the most common cause of neonatal death (7). In 2021, neonatal mortality remained high (27 per 1000 live births) despite the nation's target of 15 per 1000 live births (7). Local studies have shown that inadequate skills among health-care workers largely contribute to this finding (8). In order to reduce mortality, standardization of neonatal resuscitation across the country is paramount. Thus, we aimed to develop a neonatal resuscitation algorithm for a resource-limited country (Zambia) which reflects the diversity of newborn life support situations.

## Algorithm

### Helping babies breathe (HBB)

The HBB algorithm is a basic resuscitation algorithm developed for limited resource settings (2–4, 9). Most newborn infants transition from intra- to extra-uterine life with no or only little support while 3%–6% require bag-and-mask-ventilation (BMV) and <1% requires advanced neonatal resuscitation involving chest compression and drug administration (10, 11). Thus, the HBB algorithm highlights BMV as the crucial part of neonatal resuscitation (10). In case no chest rise is seen, the infant's head is repositioned and visible secretions are suctioned but no other airway maneuvers are performed in case the above interventions fail. If the chest rises with manual ventilation but the heart rate does not rise, ventilation should be continued until help arrives (4).

HBB is appropriate in settings where advanced resources and skills are unavailable. In Zambia, the period following the implementation of HBB saw a reduction in mortality from 34 to 24 per 1000 live births over a ten-year period, and HBB is still taught in midwifery schools (12).

### Newborn life support (NLS) algorithm

The NLS algorithm instructs to give five inflation breaths in non-breathing infants. This may assist in creating functional residual capacity (FRC) after birth (13, 14). In case of insufficient chest rise, the algorithm also suggests to reposition and suction but also advises to place an oropharyngeal airway or use a jaw thrust. After the initial five inflation breaths, ventilation breaths are administered.

The NLS algorithm considers the assessment using color, tone, breathing, and heart rate at various points in the algorithm (15). While color may not be a good indicator of low oxygen saturations in dark-skinned babies (16), it may also be particularly useful in resource-limited settings where pulse oximetry is not readily available. Additionally, although the other indicators are universally acknowledged to be signs of neonatal cardiopulmonary adaptation, accurate heart rate assessment may be challenging in resource-limited countries (17).

Chest compressions are recommended if the heart rate remains <60 beats per minute and if chest compressions are unsuccessful, adrenaline should be administered. Ten percent dextrose is given in prolonged resuscitations to reduce the likelihood of hypoglycemia, repeated saline volume boluses in case of suspected hypotension and sodium bicarbonate is considered in prolonged resuscitation with adequate ventilation to reverse intra-cardiac acidosis. Normal saline can be replaced by O negative blood in case of infant anemia (15).

### Zambian neonatal resuscitation algorithm

**Figure 1:**
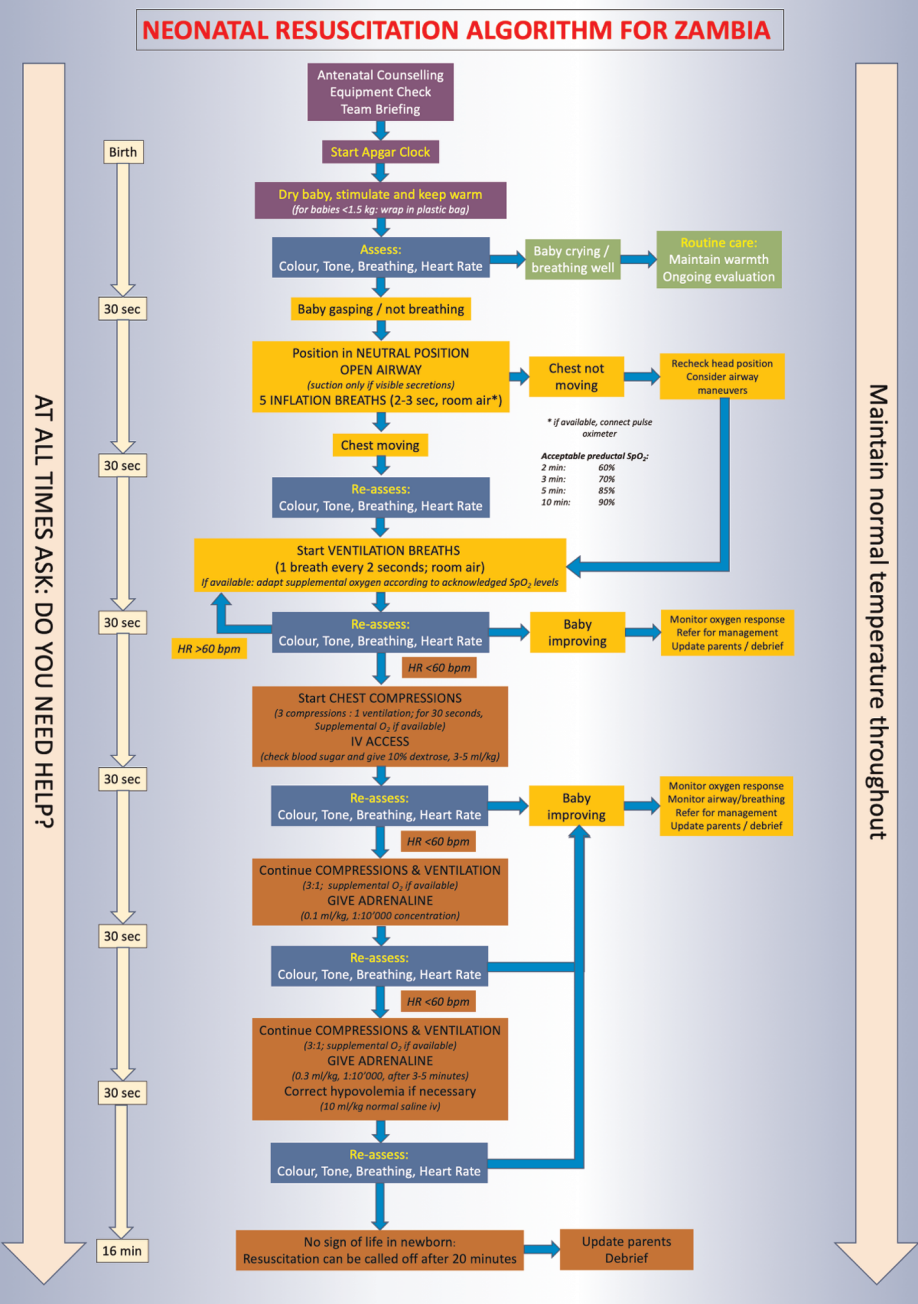
Zambian neonatal resuscitation algorithm. Colour code of the boxes: Violet = initial steps for all newborn infants Blue = assessment of infants Green = routine care in a baby not requiring resuscitation Yellow = respiratory support and monitoring Red = cardiovascular support and end of resuscitation efforts.

New advances in health care provision have improved the availability of operative deliveries in remote spaces and consequently, more level I and level II neonatal units are planned to be built over the next five years. Thus, there is the need for skills in advanced neonatal resuscitation even in more remote places in order to stabilize and transfer patients to level III institutions. Based on these considerations, we developed a home-grown neonatal resuscitation algorithm that caters to all levels of care. This algorithm was designed by people who are familiar with the country's specific needs. The group consists of clinicians from various specialties including anaesthetists, midwives, nurses, paediatricians, and neonatologists who came together for regular meetings to discuss the ideal approach to neonatal resuscitation in Zambia. The final version incorporates ideas from the HBB and the NLS algorithm and was generated in an iterative process through group discussions among the members of this working group. Figure 1 displays the final version of the Zambian neonatal resuscitation algorithm.

The algorithm takes into account antenatal counseling and identification of risk factors of babies that may require neonatal resuscitation. Women with risk factors are counseled to have their regular antenatal check-ups in facilities prepared to perform operative deliveries. Preparation for delivery is key and the need to have a neonatal resuscitation team available for births with risk factors is stressed. Ideally, each team should include three persons (group leader in charge of the airway, assistant for heart rate assessment and someone documenting and handing over equipment if needed). Room preparation includes maintaining a room temperature of 24–26 °C and ensuring that windows and doors are closed to avoid drafts. Food grade plastic bags are used and pulled up to the neck in babies <1.5 kilograms who are not dried post delivery. All babies, regardless of gestational age should be placed under a radiant warmer and a hat should be placed on their heads to prevent hypothermia.

Resuscitation commences with drying and stimulating the baby while simultaneously assessing color, tone, breathing and heart rate. Cord clamping is delayed for 1 to 3 min for infants who do not require resuscitation. Immediate cord clamping is performed in case resuscitation is needed.

In non-breathing infants, the airway is assessed with the head placed in neutral position. If secretions are visible, suctioning is performed with a penguin sucker or suction machine, depending on availability. Importantly, suctioning should not be prioritized over giving inflation breaths. Assessment using color, tone, breathing and heart rate is done every 30 s with a focus on breathing and heart rate as the most important contributors to decision-making during resuscitation. Color and tone are mainly important in situations where breathing is difficult to evaluate (e.g., during mask ventilation). Heart rate is determined using a stethoscope and the examiner tapping on the bed to alert the team of the heart rate. While there is some inaccuracy using this technique (17), it is readily available in all situations across the country and is thus prioritized as primary mode of heart rate evaluation.

In continuously non-vigorous infants, inflation breaths are delivered at a pressure of approximately 30 cmH_2_O lasting 2–3 s for each breath using a self-inflating bag and mask. Chest rise is assessed during the inflations. If the infant remains floppy, airway maneuvers should be performed with emphasis on the oropharyngeal airway. Meconium-stained liquor warrants laryngeal suctioning under direct visualization only if the chest is not rising during inflation breaths. Ventilation breaths of 1–2 s duration are performed after the initial inflation breaths have been delivered, thus achieving the infant's inherent respiratory rate.

If the infant's HR remains <60 bpm after at least one minute of ventilation, oxygen is increased to 100% (where available) and chest compressions are started in a ratio of 3 chest compressions to 1 ventilation breath. Simultaneously, a peripheral or umbilical catheter access is sought to administer 10% dextrose. Reassessment of HR is done every 30 s. If HR remains <60 bpm, adrenaline is given at 0.1 ml/kg of 1 in 10,000 concentrations. A fluid bolus (10 ml/kg regular saline) is given if the capillary refill time remains ≥3 s. Administration of Sodium bicarbonate is not recommended in the algorithm. Resuscitation is terminated after 20 min if there is no response to resuscitative efforts.

Intubation equipment is only available in few hospitals and, as unsuccessful intubation may reduce the time of adequate ventilation, the algorithm does not explicitly recommend intubation. However, in case an experienced intubator is available, it can be performed on a case-by-case basis. In facilities without intubation and ventilation facilities, patients should be transferred as soon as possible. Until then, ventilation should be continued, and oxygen administered if possible.

## Discussion

Neonatal mortality remains high in most limited-resource countries and continues to cause concern. In Zambia, neonatal mortality rates are among the highest in Sub-Sahara Africa. In order to achieve the United Nations Sustainable Development Goal (SDG) number 3.2 (lowering neonatal mortality below 12 per 1000 live births) (18), concerted efforts to improve quality of care are required. Thus, we developed a new neonatal resuscitation algorithm catering to different situations across Zambia, thus bridging the gap between rural limited-resource situations to better-equipped level III centers in the same country.

A bottleneck analysis by the World Health Organization showed that various resources need to be improved in Zambia to reduce neonatal mortality (19). Advancement of skills is most important as a large percentage of neonatal deaths may be prevented without sophisticated equipment. Further low cost interventions (e.g., antenatal steroids, use of plastic bags, kangaroo mother care, early breast milk feeding, intravenous fluids, use of antibiotics and use of continuous positive airway pressure) are measures that can be employed easily to reduce deaths related to prematurity (20, 21). Low cost interventions like Kangaroo Mother Care (KMC) are ideal for low resource setups with inadequate incubators and have been shown to reduce mortality due to prematurity (21–23).

Intrapartum monitoring and preparation are key but most importantly, health-care workers need to be skilled in performing neonatal resuscitation. Lack of neonatal resuscitation skills is a significant cause for continuously high rates of morbidity secondary to birth asphyxia (7). This finding may be partly caused by the use of different resuscitation algorithms throughout the country, thus rendering resuscitation training more difficult. In order to harmonize and facilitate team work, a resuscitation algorithm was needed which could be used at different levels of care. The roll-out of this algorithm also includes teaching health-care workers from different levels of care in the same sitting.

Unlike the HBB algorithm, where only bag-and-mask-ventilation is performed and which only includes repositioning of the head and mask if no chest rise is noted, our algorithm aims to go one step further by training health-care workers in placing an oropharyngeal airway in a floppy infant. Placing an oropharyngeal airway is relatively easy to learn and may decrease the rate of failed resuscitations. Intubation is not emphasized on in order to avoid any delay in ventilation times.

Giving inflation breaths improves functional residual capacity (14), and is recommended in our algorithm. While sustained inflations may cause harm in extremely preterm infants (24), only infants ≥28 completed weeks gestation are cared for in Zambia and the inspiratory time used in our algorithm is much shorter than the i-time used in the respective trial (24).

The algorithm is currently rolled out across Zambia in order to unify neonatal resuscitation across the country. Before nation-wide roll-out, it was implemented and tested in the largest neonatal referral centre, the University Teaching Hospital in Lusaka, Zambia. Here, the algorithm was first introduced and students and young doctors specializing in paediatrics or neonatology were trained on the neonatal unit. Subsequently, the algorithm was tested in the delivery room where implementation was successful. The test phase was important to realise potential pitfalls in teaching the algorithm, improved the algorithm and allowed for a more successful nation-wide roll-out. Thus far, 500 health-care workers from over 30 facilities (in 5 of the 10 provinces) have been trained. Trainers travel to the more centrally located health care facilities for the training which consists of a five-day training. Participants are equipped with knowledge and skills in conducting neonatal resuscitation and post-resuscitation care, as well as safe transfer of a neonate in a multidisciplinary five-days training including all staff likely to attend deliveries. This encourages teamwork and task shifting. Assessment and management of newborn infants is taught including respiratory support, calculation of fluids, and temperature management. Since CPAP devices are not ubiquitously available, both regular CPAP and an improvised CPAP using a 500 ml plastic bottle are taught. Refresher courses will be provided every two years to ensure a sustained gain of knowledge. The roll-out will be continuously monitored and feedback from the participants implemented. A train-the-trainer approach is utilized, thereby ensuring a consistent transfer of knowledge. This algorithm will be rolled out in all training institutions and delivery facilities across Zambia over the next months.

## Conclusion

The Zambian neonatal resuscitation algorithm is the first algorithm crafted by experts working in Zambia, making it a suitable algorithm for all levels of care in this country. This home-grown neonatal resuscitation algorithm is likely to bridge the gap between limited situations requiring only bag and mask ventilation and better equipped institutions where more advanced resuscitation is possible.
